# Economic burden of major depressive disorder: a case study in Southern Iran

**DOI:** 10.1186/s12888-022-04220-7

**Published:** 2022-08-30

**Authors:** Khosro Keshavarz, Arvin Hedayati, Mojtaba Rezaei, Zahra Goudarzi, Ebrahim Moghimi, Mehdi Rezaee, Farhad Lotfi

**Affiliations:** 1 Health Human Resources Research Center, School of Health Management and Information Sciences, Shiraz University of Medical Sciences, Shiraz, Iran; 2 Emergency Medicine Research Center, Shiraz University of Medical Sciences, Shiraz, Iran; 3 Department of Psychiatry, School of Medicine, Research Center for Psychiatry and Behavior Science, Ebnesina Hospital, Hafez Hospital, Shiraz University of Medical Sciences, Shiraz, Iran; 4 Student Research Committee, School of Management and Medical Information Sciences, Shiraz University of Medical Sciences, Shiraz, Iran; 5 Health Human Resources Research Center, School of Health Management and Information Sciences, Shiraz University of Medical Sciences, Shiraz, Iran; 6 Department of Psychiatry, School of Medicine Research Center for Psychiatry and Behavior Science Ebnesina Hospital, Shiraz University of Medical Sciences, Shiraz, Iran; 7 Department of Health Management, Policy and Economics, Faculty of Management and Medical Information Sciences, Kerman University of Medical Sciences, Kerman, Iran

**Keywords:** Economic burden, Major depressive disorder, Direct medical costs, Direct non-medical costs, Indirect costs

## Abstract

**Background:**

Depression disorders are a leading cause of disability in the world which imposes a significant economic burden on patients and societies The present study aimed to determine the economic burden of Major Depressive Disorder (MDD) on the patients referred to the reference psychiatric single-specialty hospitals in southern Iran in 2020.

**Methods:**

This cross-sectional research is a partial economic evaluation and a cost-of-illness study conducted in southern Iran in 2020. A total of 563 patients were enrolled through the census method, and a researcher-made data collection form was used to gather the required information. The prevalence-based and the bottom-up approaches were also used to collect the cost information and calculate the costs, respectively. The data on direct medical, direct non-medical, and indirect costs were obtained using the information in the patients’ medical records and insurance bills as well as their self-reports or those of their companions. To calculate the indirect costs, the human capital approach was used as well.

**Results:**

The results showed that the annual cost of MDD was $ 2717.41 Purchasing Power Parity (PPP) (USD 2026.13) per patient in 2020. Direct medical costs accounted for the largest share of the costs (73.68%), of which hoteling and regular beds expenses were the highest (57.70% of the total direct medical costs). The shares of direct non-medical and indirect costs were 7.52 and 18.80%, respectively, and the economic burden of the disease in the country was estimated at $7,120,456,596 PPP (USD 5,309,088,699).

**Conclusion:**

In general, due to the high prevalence of MDD and the chronicity of the disease, the costs of its treatment can impose a heavy economic burden on the society, healthcare system, insurance system, and the patients themselves. Therefore, it is suggested that health policymakers and managers should take appropriate measures to increase the basic and supplemental insurance coverage of these patients. In addition, in order to reduce the costs, proper and equitable distribution of psychiatrists and psychiatric beds, expansion of home care services, and use of Internet-based technologies and the cyberspace to follow up the treatment of these patients are recommended.

**Supplementary Information:**

The online version contains supplementary material available at 10.1186/s12888-022-04220-7.

## Introduction

Major depression, also known as major depressive disorder (MDD) or clinical depression, is a mood disorder that causes a constant feeling of sadness and loss of interest [1]. Major depression affects how a person feels, thinks, and behaves, and may lead to a variety of emotional and physical problems [2]. Thus, the patient might have difficulty doing normal daily activities and feel that life is worthless [2–4]. Depression is globally recognized as the main cause of years of life lost due to disability (YLDs) and illness [5]. Over 300 million people worldwide suffer from depression, accounting for about 4.4% of the world’s population [6].

MDD often begins in adolescence or at the age of 20–30, but it can generally occur at any age. It is more common among women, perhaps because women are more likely to seek treatment [7].

According to studies, the prevalence of depression in different Iranian nations has been varied up to 73% [8]. In addition, the prevalence of this disease has been higher in rural population and small towns than in large cities [9]. In 2017, MDD affected approximately 163 million people (2% of the world’s population) [10]. The percentage of people affected by major depression at some point in their lives varies from 7% in Japan [11] to 21% in France [12]. The prevalence rate of depression is higher in developed countries (15%) compared to developing ones (11%) [13]. A research conducted in 2019 showed that the economic burden of MDD treatment in the United States was between $8662 and 16,367 a year, depending on the severity of the disease. In other words, the more sever the disease, the heavier its economic burden would be [14]. Furthermore, according to the studies by the World Health Organization, depression and anxiety added up to a total of over $ 1 trillion in economic losses each year, while the global cost of mental disorders was estimated at about $2.5 trillion in 2010. It is estimated that this amount will reach $ 6 trillion by 2030 with a growth rate of 240%. It is noteworthy that as the most common mental disorders, depression and anxiety will definitely make up a significant share of these figures [15]. In general, MDD is the fourth disease in Iran in terms of disease burden [16]. Given these statistics, there is no doubt that depression should be taken into account as a priority by health system policymakers and decision makers. Optimal allocation of limited resources to unlimited needs in the health area necessitates studies to be conducted on economic evaluation. However, relying on the evidence from global studies will not guarantee the success of national policies and planning at the national level. Thus, even in the presence of such global studies, the need for conducting national research is evident [17]. Health costs have risen dramatically around the world in recent decades. The healthcare system in Iran has faced significant challenges due to the rising costs of healthcare services. Due to limited resources to provide healthcare services, effective provision of such services is one of the common concerns of healthcare systems around the world [18]. The review of the literature indicated that, no study had been specifically conducted on the economic burden of MDD in Iran. Given the high prevalence of depression and its significant burden on the patients, the health system, and society, it seems necessary to apply appropriate methods of risk factors identification, prevention, treatment, and management of this disease. Furthermore, MDD increases the economic costs imposed on the individuals and society due to various reasons such as urgency to leave work and not payment, and the need to be admitted to psychiatric treatment centers. Therefore, this study was conducted to determine the economic burden of MDD in Iran and deliver an evidence to policy makers for prioritizing different programs.

## Materials and methods

This partial economic evaluation is a cost-of-illness study conducted through a cross-sectional method in 2020 in Fars province. The province in south of Iran with more than 5 million populations, is a referral province for patients. It has 55 governmental hospitals under the coverage of the Shiraz, Fasa, Jahrom, Larestan and Gerash universities of medical sciences that two of them including Ostad Moharrari and Ibn Sina hospitals are single specialty hospitals in psychiatry disorders area.

The study population consisted of all MDD patients who referred to these hospitals in 2020 and hospitalized there. According to the statistics of these centers, the total number of the MDD patients was 563, and they were examined through census.

Furthermore the costs were collected using a researcher-made cost collection form as well as the opinions of psychiatrists and health economists and the patients’ self-declarations. The data collection form consisted of four sections: demographic characteristics of the patients, including age, sex, marital status, education level, employment status, type of basic health insurance coverage, supplemental insurance coverage, place of residence, and the mean monthly income.

In this study, the social perspective approach was used to extract the costs, i.e. direct medical costs (DMC), direct non-medical costs (DNMC), and indirect costs (IC), which are discussed below.

### Inclusion and exclusion criteria

Inclusion criteria included all patients referred to referral hospitals in Shiraz who their disease had been diagnosed MDD based on DSM-IV diagnostic criterion by physicians. Also, having a comorbidity and unwillingness to participate in the study regarding to consent form were exclusion criteria.

### Direct medical costs

DMC of each patient were retrospectively determined and collected using a researcher-made checklist by referring to the reference hospitals under study. These costs included visits, medications, tests, radiology, convulsive therapy, hoteling services, etc., determined and extracted separately from the patients’ medical and financial records as well as the hospital information system in 2020 in order to increase the accuracy of the data.

### Direct non-medical costs

DNMC were obtained using a cost-collection form and through interviews with the patients. The DNMC included the costs of travelling to the centers to receive medical services as well as accommodation and meal expenses of the patients and their companions.

### Indirect costs

IC included absenteeism and lost productivity of the patients and their companions, which were calculated for each patient based on their average daily income lost due to absence from work for hospitalization or treatment follow-up, the average daily income lost for each patient’s companion due to absence from work to accompany or care for the patient. The calculation process was done through interviews with the patients and based on the human capital approach [19]. In the present study, the patients’ wages were used to calculate the lost income. In addition, the minimum daily wage determined by the Ministry of Labor was used for the housewives and students aged 15–65 ($ 19.53 Purchasing Power Parity (PPP), equivalent to USD 14.56 as the average daily wage in 2020) [20]. Eight working hours was considered one working day.

For increasing the accuracy and precision of data, we tried to obtain the opinions of patients, accompanying patients, specialist and medical records simultaneously, Also, in order to examine the uncertainty data, sensitivity analysis was used.

Also, regarding to importance of data collection quality, firstly, we extracted data from patients’ records and hospital information system (HIS); Secondly, for collecting outpatient data, we interviewed with patients and their accompanying. In order to ensure their accuracy and validity, the experts’ opinions were included. Also, for preventing recall error in indirect and direct non health costs, the recent 3 months patients information were excluded and then multiplied by 4 for estimating yearly total cost.

### Calculation of economic burden of major depression

In this study, the prevalence-based and bottom-up approaches were used to calculate the economic burden and costs. The former approach is used when the patient’s costs in a period of usually 1 year are measured as the costs of the study year, and in the latter, the resources used by each person are measured. Thus, the bottom-up method is able to differentiate treatment differences between the patients [21]. It should be noted that in this study, all the costs were converted into dollars using the PPP exchange rate as each dollar equal to 31,317 Rials and the reference currency index as each USD equal to 42,000 Rials in 2020 [22]. The data on the prevalence in the country were needed for estimating the number of MDD patients. According to previous studies, the prevalence of this disease was 4.1% in the population over 15 years of age [23, 24].

Therefore, considering the population of 63,910,000 people over 15 years of age in the country in 2020 [25], the total number of MDD patients in Iran was estimated 2,620,310 people. Finally, after collecting the data on the prevalence rate, population, and the mean cost per patient, the economic burden of the disease in the country was calculated using the following formula [26]:

$$\mathrm{Economic}\;\mathrm{burden}:\;\mathrm{Total}\;\mathrm{cost}\;(\mathrm{Direct}\;\mathrm{Medical}\;\mathrm{Cost}\;+\;\mathrm{Direct}\;\mathrm{Non}-\mathrm{medical}\;\mathrm{Cost}\;+\;\mathrm{Indirect}\;\mathrm{Cost})\;\ast\;\mathrm{estimated}\;\mathrm{number}\;\mathrm{of}\;\mathrm{infected}\;\mathrm{inpatient}\;\mathrm{cases}\;\mathrm{in}\;\mathrm{Iran}$$

The Excel 2016 software was used for data analysis.

### Sensitivity analysis

To conduct the one-way sensitivity analysis, a study carried out in the country in which a prevalence of 4.1% (95% CI: 3.1–5.1%) had been reported was used [23, 24]. Hence, 3.1 and 5.1% were considered as the low and high prevalence limits, respectively.

Besides, In order to ensure the quality of study, Drummond checklist [27] and checklist parts related to cost quality were used. The Word file is in Appendix 1.

## Results

### Demographic characteristics

A total of 563 patients participated in this study, their demographic characteristics are presented in Table 1. The results showed that most of the patients were male (71.4%), unemployed (67.32%), and married (47.78%), had secondary school or lower education levels (91.47%), were residents of Fars province (89.70%), covered by an insurance (96.45%), did not have supplemental insurance (84.2%). In addition, most of the patients were 25–44 years of age (57.73%).

**Table 1:** Demographic characteristics of the patients with Major Depressive Disorder southern Iran in 2020

Variable		patients with Major Depressive Disorder	
		Number	%
Sex	Male	402	71.4
	Female	161	28.6
Age	≤15	2	0.35
	15–24	64	11.37
	25–44	325	57.73
	45–64	156	27.71
	≥65	16	2.84
Marital status	Single	269	47.78
	Married	234	38.02
	Divorced	80	14.2
Education level	Secondary school or lower	515	91.47
	Academic	48	8.53
Employment status	Employed	168	29.84
	Retired	16	2.84
	Unemployed	379	67.32
Residence	Fars Province	505	89.70
	Other provinces	58	10.30
Basic insurance	Insured	543	96.45
	No insurance	20	3.55
Supplemental insurance	Yes	89	15.8
	No	474	84.2

In Ibn Sina Hospital, based on specialty’s opinion and medical records, the average number of medications for MDD patients was 4 items (Sodium Valproate, Olanzapine, Sertraline, Citalopram) in first 6 months and 2 items (Sodium Valproate, Citalopram) in second 6 months per day.

Also, In Ostad Moharrari Hospital, the average number of medications was 3 items (Sodium Valproate, Olanzapine, Sertraline) in first 6 months and 2 items (Sodium Valproate, Olanzapine) in second 6 months.

The average long of stay for these patients in Ibn Sina and Ostad Moharrari was 15.44 and 20 days respectively. Also, the total occupied bed was 4880 and 5164.

### Direct and indirect costs

Table 2 shows the mean costs of the patients with major depressive disorder. The mean DMC per patient was $ 2002.08 PPP (USD 1492.82) and the DNMC was $ 204.52 PPP (USD 152.44) as well. In addition, the cost of hoteling and regular bed was $ 1155.3 PPP (USD 861.44), accounting for the highest share of DMC (57.7%), and the patient’s companion’s travel cost was $ 98.24 PPP (USD 73.25), accounting for the highest share of DNMC. Furthermore, the mean IC per patient was $ 510.81 (USD 380.87), the largest share of which was the cost of absenteeism (84.7%) at $ 432.67 PPP (USD 322.61).

**Table 2:** Mean direct medical, direct non-medical and indirect costs per patient with Major Depressive Disorder in 2020 from a social perspective

Type of cost		PPP	USD	%	Total cost (%)
Direct medical costs	Hoteling	1155.3	861.44	57.70	73.68
	Visit	568.57	423.95	28.40	
	Consultation services	4.1	3.06	0.20	
	Nursing services	69.69	51.96	3.48	
	Medicine	26.6	19.83	1.33	
	Consumables	2.17	1.62	0.11	
	Rehabilitation	1.37	1.02	0.07	
	Computed Tomography Scan (CT scan)	0.56	0.42	0.03	
	Barograph	2.02	1.5	0.10	
	Laboratory	72.55	54.1	3.62	
	Radiology	2.47	1.84	0.12	
	Sonography	0.39	0.29	0.02	
	Magnetic Resonance Imaging (MRI)	0.42	0.31	0.02	
	Electroconvulsive therapy (ECT)	56.54	42.16	2.82	
	Echocardiography	0.07	0.05	0.003	
	Psychology services	3.75	2.79	0.19	
	Other services	35.51	26.48	1.77	
	Total	2002.08	1492.82	100	
Direct non-medical costs	Patients Transportation	73.68	54.93	36.03	7.52
	Patient’s commute Transportation	98.24	73.25	48.03	
	Patient’s accommodation and food	13.97	10.41	6.83	
	accommodation and food for Patient’s commute	18.63	13.85	9.11	
	Total	204.52	152.44	100	
Indirect costs	Missed workdays due to illness	432.67	322.61	84.70	18.80
	patient companions’ missed workdays due to patient care	78.14	58.26	15.30	
	Total	510.81	380.87	100	
	Total cost	2717.41	2026.13	100	

*CT scan* Computed Tomography Scan; *MRI* Magnetic Resonance Imaging; *ECT* Electroconvulsive therapy

### Economic burden of costs for MDD in Iran

**Fig. 1:**
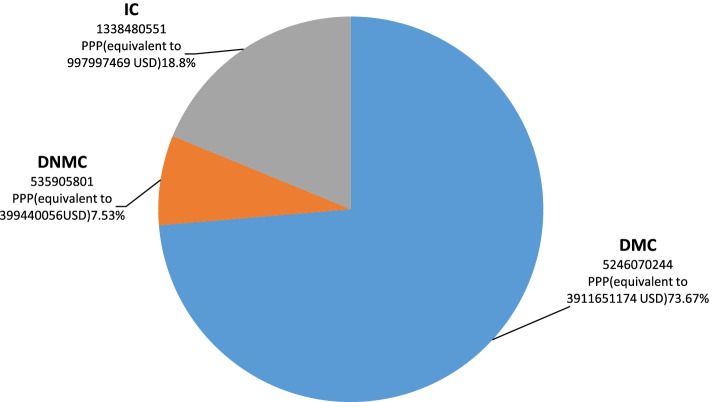
Estimation of annual economic burden of major depressive disorder in Iran in 2021 from a social perspective ($ PPP and USD)

Given the number of patients in the country estimated by the prevalence of the disease, and based on the mean costs extracted from the results of this study, the estimated economic burden for all MDD patients in the country is presented in Table 3. Accordingly, the mean annual cost of the patients with major depressive disorder in Iran in 2020 was $7,120,456,596 PPP (equivalent to USD 5,309,088,699). The results also showed that DMC (73.67%) accounted for the highest economic burden of MDD in the country ($ 5,246,070,244 PPP equivalent to USD 3,911,651,174). Figure 1 displays the total mean DMC, DNMC, and IC of MDD in Iran in 2020.

**Table 3:** Estimation of total annual costs of patients with major depressive disorder in 2020

Number of patients	Direct treatment costs			Direct non-medical costs			Indirect costs			Economic burden		
	PPP	USD	%	PPP	USD	%	PPP	USD	%	PPP	USD	%
**2,620,310**	5,246,070,244	3,911,651,174	73.67	535,905,801	399,440,056	7.53	1,338,480,551	997,997,469	18.80	7,120,456,596	5,309,088,699	100

### Sensitivity analysis

To do the one-way sensitivity analysis, the number of MDD patients was considered variable according to the low and high prevalence limits reported in Iran, and the cost components per patient were assumed constant. The total cost was then calculated, the results of which are presented in Table 4.

**Table 4:** Sensitivity analysis for DMCs, DNMCs, ICs, and total costs of MDD patients in Iran in 2020

One-way Sensitivity analysis (PPP)	Number of patients in Iran	DMC		DNMC		IC		COI	
		PPP	USD	PPP	USD	PPP	USD	PPP	USD
Lower limit	1,981,210	3,966,540,916	2,957,589,912	405,197,069	302,015,652	1,012,021,880	754,583,452	5,383,759,865	4,014,189,016
Upper limit	3,259,410	6,525,599,572	4,865,712,436	666,614,533	496,864,460	1,664,939,222	1,241,411,486	8,857,153,327	6,603,988,382

*DMC* Direct Medical Costs; *DNMC* Direct Non-Medical Costs; *IC* Indirect Costs

## Discussion

The rising costs of health systems around the world, especially in low- and middle-income countries such as Iran, have become a major concern for health managers and policymakers, the main reason for which is the increasing number of non-communicable and chronic diseases such as major depressive disorder. The medical costs of the MDD patients have increased rapidly in recent years [28]. The present study aimed to calculate the costs of MDD for the patients referred to the reference medical centers in southern Iran and determine the economic burden of the disease in the country in 2020.

As to demographic characteristics, the results of this study showed that most of the patients were male, were of 25–44 years of age, were married, had under-diploma education levels, were unemployed, had a health insurance, were not covered by supplemental insurance, were residents of the cities in Fars province, and had no income. The results of the study by Pourahmadi et al. (2019) on the patients with depression in Iran showed that most of them were male, were averagely aged 41 years, and were covered by a health insurance [29].

In terms of the costs, the results of the present study indicated that the economic burden of MDD was $ 7,120,456,596 PPP. Cheng et al. (2012) in South Korea estimated the economic burden of depression at $ 4049 million, with the IC, DMC, and DNMC accounting for the highest shares, respectively ($ 3880 million, $152.6 million, and $15.9 million, respectively) [30]. This is consistent with the results of the present study in terms of the total economic burden of the disease in the study year, but is not consistent in terms of indirect costs. One of the reasons for such a discrepancy could be the calculation of intangible costs.

The economic burden of medical direct cost of MDD was approximately 7% of the total healthcare costs in 2020. The total cost of the health system in Iran was $512 billion PPP in 2020, accounting for 7% according to the gross domestic product (GDP) that was $1.69 trillion PPP in that year as reported by the International Monetary Fund [31, 32].

As stated in this study, the mean cost of MDD was $ 2717.41 PPP per patient, which is in line with the results of the studies by Zhdanava et al. (2021) in the United States [33], Tanner et al. (2020) in Canada [34], and Zaprutko et al. (2019) in Poland [35] in terms of the mean cost of the disease.

The results also showed that DMC accounted for the largest share of the total cost of MDD treatment (73.67% of the total cost of the disease), implying that DMC was the most important cost component for the patients with major depressive disorder. Furthermore, the largest share of DMC was related to hoteling and regular beds of these patients (57.70%), which could be due to the high average length of stay (number of hospitalization days), and the cost of service provision per hospital bed in Iran. Pourahmadi et al. (2019) in Iran calculated the economic burden of depression and suggested that hoteling services accounted for the highest cost of the patients (62%) [29], which is consistent with the results of the present study.

Olchanski et al. (2013) examined the economic burden of refractory depression and showed that the disease was associated with significant medical costs per patient due to greater use of care and treatment, and the costs increased significantly with increasing disability. Moreover, DMC was one of the most important cost components for the patients suffering from depression [36]. In Poland, Zaprutko et al. (2019) conducted a study on 548 patients with depression and showed that direct costs accounted for the largest percentage of the costs [35]. In addition, Ruggeri et al. (2022) in Italy studied the patients with depression and stated that direct costs accounted for a high percentage of the total costs [37].

However, Cheng et al. (2012) carried out a study on economic burden of depression in South Korea and found that DMC accounted for the lowest percentage of the total costs. They estimated the total cost of depression at $ 4049 million, of which $ 152.6 million belonged to DMC. On the other hand, the IC was estimated at $ 3880.5 million. They concluded that, especially in terms of disability, depression imposed a significant burden on the society and patients, and societies should strive to increase public health in order to prevent and diagnose depression to ensure that appropriate treatment would be provided. Such measures might lead to a significant reduction in the overall burden of depression [30].

In their study aiming at examining the economic burden of MDD on the adults in the United States from 2005 to 2010, Greenbergh et al. (2015) found that DMC accounted for the lowest percentage of the total economic burden on the adults with major depression, which is not consistent with the results of the present study [38]. This can be due to the provision of more services in outpatient care centers and lower costs of hoteling and hospitalization of the patients in these countries.

The present study also showed that DNMC had the lowest share of the total treatment costs, accounting for 7.53% of the total costs. The results of this study are not consistent with those of Cheng et al. (2012) in South Korea and Tanner et al. (2020) in Canada who concluded that DNMC accounted for the highest percentage of the total costs, and travel expenses accounted for a small share [30, 34]. The reasons for this discrepancy could be the extent to which aids were available to adapt to the environmental conditions at home and work, and the cost of purchasing them varied in the countries under study.

Greenberg et al. (2021) in the United States also found that DNMC accounted for less than 4% of the total costs and therefore, had the lowest share of the costs for MDD patients [39]. The reason for this discrepancy could be the difference in the number of non-native patients in these studies as well as the difference in travel and accommodation costs in the countries studied.

In this study, IC accounted for 18.8% of the total cost of patient treatment, which is in line with the results of the study by Pour Ahmadi et al. (2019) [29].

However, the results of a study by Sobocki et al. (2007) on the economic burden of depression in Sweden from 1997 to 2005 showed that the total cost of depression increased from € 1.7 billion in 1997 to €3.5 billion in 2005. In other words, the economic burden of depression was doubled in the society, while direct costs were relatively stable over time. In 2005, indirect costs accounted for € 3 billion (86% of the total costs), which is inconsistent with the results of the present study, perhaps due to the high daily wages of the patients in these countries [40].

The results of the studies by Cheng et al. (2012) in South Korea [30], Greenberg et al. (2015) in the United States [38], Sobocki et al. (2007) in Sweden [40], and Tanner et al. (2020) in Canada [34] showed that IC accounted for a relatively high percentage of the total costs.

Regarding to increase the prevalence rate in psychiatry disorders and the high cost for their treatment during the last decade, the government should focus on prevention this type of diseases. These diseases have several causes, one of the most important causes is socio-economics problem that need to governments’ effort through programming and problem solving. Also, the ministry of health should foresight the hospital beds and in order to financial protection, health insurance organizations must participate in share of costs greatly.

One limitation of the present study was the self-declaration of the patients or their companions about DNMC and IC, as they were likely to forget or approximate recall some of the costs (recall bias). Another research limitation, defect information in some patients’ medical records including prescribed medicines Furthermore, intangible costs were not calculated in this study due to the impossibility of measuring them accurately.

Moreover, Due to lack of some patients’ income information, we used minimum wages of labor department and for improving the process of cost estimation, the sensitivity analysis was done.

The number of patients was another limitation in finding generalizability. Although, we include almost all patients with major depressive disorder.

## Conclusion

In general, due to the high prevalence of MDD in Iran and the chronicity of the disease as well as the need for lifelong treatment, the costs of treating this disease can impose a heavy economic burden on the society, the healthcare system, the insurance system, and the patients themselves. According to the obtained results, in order to reduce the economic burden of this disease, it is suggested that health policy makers and managers try to increase the insurance coverage of the care required by MDD patients. Due to the high cost of hoteling and regular beds, the provision of such services should also be covered by supplemental insurance. In addition, in order to reduce the travel costs that accounted for the highest percentage of DNMC, the following measures are recommended to prevent unnecessary patient travel: appropriate and equitable distribution of psychiatrists and psychiatric beds, expansion of home care services for MDD patients, and use of Internet-based technologies and the cyberspace to follow up the patients’ treatment.

## Supplementary Information

**Additional file 1.**

## Data Availability

The datasets generated and analyzed during the current study are available in the Science Data Bank repository, https://www.scidb.cn/s/IF7BNv.

## Abbreviations

YLDs: Years of life lost due to disability
MDD: Major Depressive Disorder
ECO: Echocardiography
CT scan: Computed Tomography Scan
MRI: Magnetic Resonance Imaging
WHO: World Health Organization
DMC: Direct Medical Costs
DNMC: Direct Non-Medical Costs
IC: Indirect Costs
PPP: Purchasing Power Parity
GDP: Gross domestic product
