# Supplementary material for: Economic burden of major depressive disorder: a case study in Southern Iran
Source: BMC Psychiatry. 2022 Aug 30;22:577. doi: 10.1186/s12888-022-04220-7 (PMC9426032; doi:10.1186/s12888-022-04220-7)
Supplement: Supplementary file 1 — Additional file 1. [file 12888_2022_4220_MOESM1_ESM.docx]

| **Row** | **Criteria** | **Address in manuscript** |
| --- | --- | --- |
| 1 | Was the main question of the study asked in an appropriate way? | Page 4-line 12 |
| 2 | Were the competitor options presented in a comprehensive manner? | NA |
| 3 | Were evidences of the effectiveness program presented? | NA |
| 4 | Were all significant costs and relevant outcomes identified? | Page 5 |
| 5 | Were all significant costs and relevant outcomes properly measured? | Page 8 |
| 6 | Were all significant costs and relative outcomes properly valued? | Page 8 |
| 7 | Were costs and outcomes adjusted for different time? | NA |
| 8 | Were an incremental analysis of the costs and outcomes of competitor options carried out? | NA |
| 9 | Were the effects of uncertainty [sensitivity analysis] investigated for all costs and outcomes? | Page 10 |
| 10 | Were all problems related to the users of the results of the study investigated during analysis and presentation of results? | NA |
